# 

**Published:** 1897-03-20

**Authors:** 


					/
THE HOSPITAL. ABSOLUTELY PURE, therefore BEST. March 20th, 189T.
OADBURY'S WW ^ CADBURY'S
COCOA
" The standard of highest purity at present ^ , . - ^
attainable,"?Lancet. ALKALIES USED
COCOA
: P
ASQoir Western INFihuA&f ' ~ - ' ?NORf.OLKahbNORWICH HOSP.LTAJ.
- Ui.M.d UU 77 I NFIRMA&T
No. 547. Vol. XXI. [_a Newspaper!] SATURDAY,
March 20th, 1897.
[sahscription/Ienna,] pB)ICE TWOPENCE.

				

